# Unveiling the Pharmacological Potential of *Anisosciadium orientale*: An Integrative Analysis of Cytotoxicity, Molecular Docking, and Apoptotic Pathways

**DOI:** 10.3390/ijms27135767

**Published:** 2026-06-26

**Authors:** Amr S. Abouzied, Wafaa M. Fouda, Mohamed K. S. El-Nagar, Bader Huwaimel, Saad Alqarni, Ali Alghubayshi, Talal Alotaibi, May S. Alanazi, Farah A. Alanazi, Nouf Rakan Alobaid, Mohamed S. Refaey

**Affiliations:** 1 Department of Pharmaceutical Chemistry, College of Pharmacy, University of Ha’il, Ha’il 81442, Saudi Arabia; b.huwaimel@uoh.edu.sa (B.H.); s.alqarni@uoh.edu.sa (S.A.); 2 Medical and Diagnostic Research Center, University of Ha’il, Ha’il 55473, Saudi Arabia; a.alghubyshi@uoh.edu.sa (A.A.); t.alotaibi@uoh.edu.sa (T.A.); 3 Department of Pharmacology and Toxicology, Faculty of Pharmacy, University of Sadat City, Sadat City 32897, Egypt; wafaa.fouda@fop.usc.edu.eg; 4 Department of Organic and Medicinal Chemistry, Faculty of Pharmacy, University of Sadat City, Sadat City 32897, Egypt; mohamed_elnagar@pharm.aun.edu.eg; 5 Department of Clinical Pharmacy, College of Pharmacy, University of Ha’il, Ha’il 81442, Saudi Arabia; 6 College of Pharmacy, Princess Nourah Bint Abdulrahman University, Riyadh 11564, Saudi Arabia; may.ksa@hotmail.com (M.S.A.); farah-ae@hotmail.com (F.A.A.); 7 College of Pharmacy, University of Ha’il, Ha’il 55211, Saudi Arabia; nouf.r.alobaid@gmail.com; 8 Department of Pharmacognosy, Faculty of Pharmacy, University of Sadat City, Sadat City 32897, Egypt; 9 Faculty of Pharmacy, National University of Sadat City, Sadat City 32897, Egypt

**Keywords:** caspase 3, lung carcinoma, antioxidant, anticancer, essential oil

## Abstract

Cancer remains the foremost cause of death globally and presents a major obstacle to increasing life expectancy. The identification of natural anticancer agents is therefore a key research priority. Essential oils (EOs), widely used in traditional medicine, possess diverse biological activities that warrant systematic investigation. *Anisosciadium orientale* is traditionally regarded as a safe, edible herb; however, its therapeutic potential has not been extensively explored. In this study, the chemical profile of the EO obtained from the aerial parts of *A. orientale* was characterized using GC–MS analysis. Antioxidant activity was evaluated through hydrogen peroxide and ABTS radical-scavenging assays, whereas anticancer effects and underlying mechanisms were evaluated in multiple cancer cell lines using MTT cytotoxicity assays, flow cytometry, and molecular docking studies. Sixty constituents were identified in the EO, with myristicin and its isomer among the major components. The EO exhibited notable antioxidant and anticancer activities, demonstrating cytotoxicity against lung carcinoma A549 cells with an IC_50_ of 84.8 µg/mL and inducing significant apoptosis accompanied by G_2_/M cell-cycle arrest. Treatment with the EO markedly boosted levels of caspase-3, p53, Bax, and the Bax/Bcl-2 ratio, while downregulating Bcl-2 expression. Molecular docking revealed strong binding affinities of major constituents—particularly myristicin and its isomer—toward the EGFR kinase active site, suggesting a high degree of complementarity with the EGFR kinase domain. Collectively, this study represents the first comprehensive study integrating chemical profiling, in vitro cytotoxicity, mechanistic assays, and molecular docking for *A. orientale*. These findings position *A. orientale* EO as a promising scaffold for the development of natural anticancer interventions, providing a foundation for future preclinical exploration.

## 1. Introduction

Medicinal and aromatic plants have long been regarded as crucial to the field of therapeutics [1]. Due to their numerous biological and therapeutic benefits, essential oils, which are secondary metabolites derived from these fragrant and therapeutic plants, are very valuable. Abundant studies have shown that these EOs have cytotoxic, anti-inflammatory, antioxidant, antiviral, and anti-microbial properties [2,3,4]. One of the main triggers of death globally and a significant barrier to extending life expectancy is cancer. An anticipated 8.5 million new incidents of cancer and 10.4 million fatalities were reported in 2023 [5]. The development of anticancer agents from nature is among the most essential ways for battling certain forms of malignancies; nevertheless, it imposes an ongoing check of natural sources such as marine species, terrestrial plants, and seaweeds [6]. Plant-based immunomodulatory drugs represent the most comprehensive advancement in anticancer therapy [7]. Moreover, numerous studies have conveyed the powerful antioxidant properties of essential oils, which are crucial for preventing cancer development. One of the families of blooming plants available worldwide, the Apiaceae (previously Umbelliferae), has 434 genera and 3780 species [8]. Research has shown that this family is rich in a variety of phytochemicals, such as terpenoids, coumarins, flavonoids, polyacetylenes, and saponins, which have been thought to be possible sources of novel potential medicinal agents [9]. More than 760 components from different chemical classes have been found in EOs that have been isolated and classified from this family. Considerable pharmacological and nutritional benefits have been demonstrated by the identified and reported components [10,11]. Several Apiaceae family members possess a variety of biological characteristics, including cyclooxygenase inhibitory, hepatoprotective, antibacterial, and antitumor properties [9]. The majority are edible and harmless plants.

**Figure 1:**
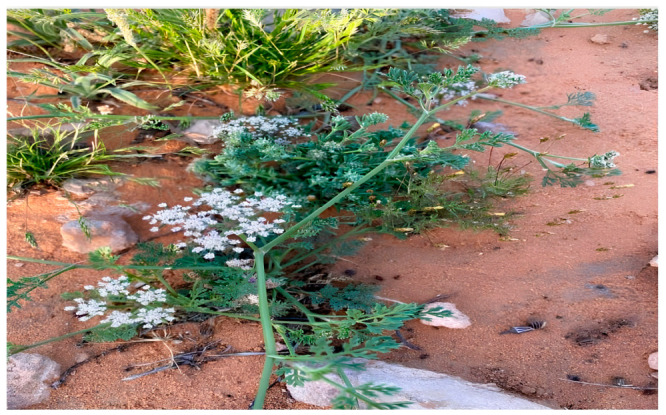
Photograph of *Anisosciadium orientale* aerial parts taken at the time of collection.

The genus *Anisosciadium* (Apiaceae) is indigenous to Southwest Asia and consists of three species: *Anisosciadium orientale* DC., *Anisosciadium lanatum* Boiss., and *Anisosciadium isosciadium* Bornm. [12]. *A. orientale* (Figure 1) is used to treat diarrhea, manage epilepsy, and relieve toothaches. Additionally, it is used as a spice or as an aromatic desert vegetable in raw, cooked, and dried forms. Even though the locals utilize it extensively in folk medicine, not much is known about this plant. The EO from *A. orientale* was analyzed in straightforward studies [12,13], although no prior papers have included in-depth examinations of the EO extracted from *A. orientale* aerial parts and its possible biological effects. As a part of our continuous efforts to explore the potential natural-derived drug templates for the treatment of cancer [6,14,15,16,17], the current study seeks to investigate the possible cytotoxic and antioxidant effects of *A*. *orientale* EO on multiple cancer cell lines, involving HL-60 (Human Leukemia-60), A549 (Lung Carcinoma Cell Line), and HePG 2 (Human Hepatocellular Carcinoma Cell Line). Furthermore, as a critical step in understanding the interaction mechanism of the EO predominant molecules as determined by GC-MS analysis, the docking investigation was conducted in accordance with the results of the biological activity.

## 2. Results and Discussion

### 2.1. Isolation and Identification of EO’s Chemical Components

**Figure 2:**
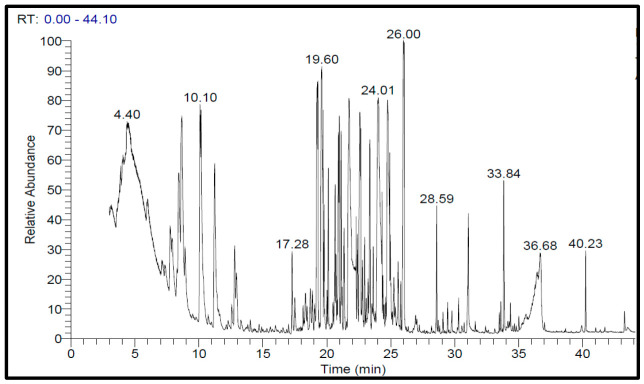
Total ion chromatogram of the essential oil of *A. orientale*.

**Figure 3:**
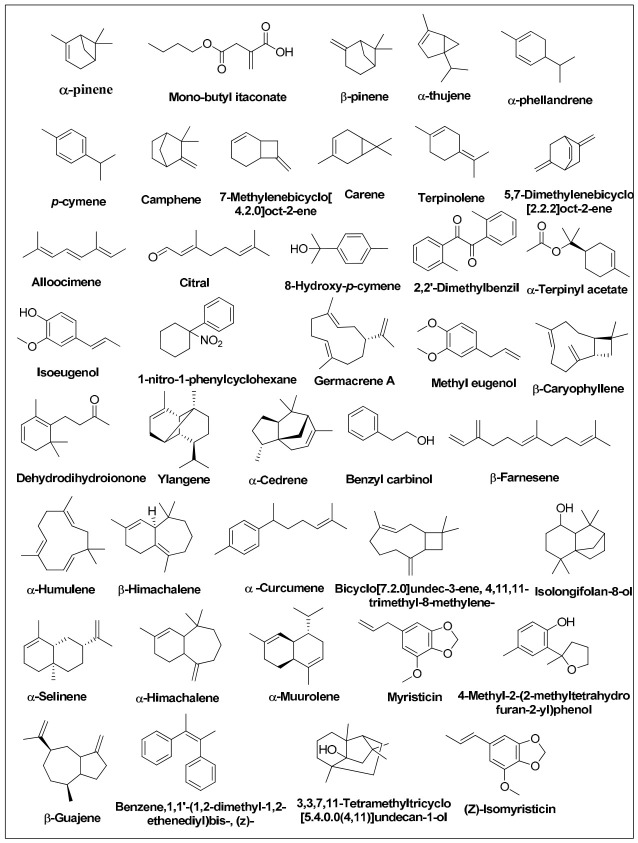

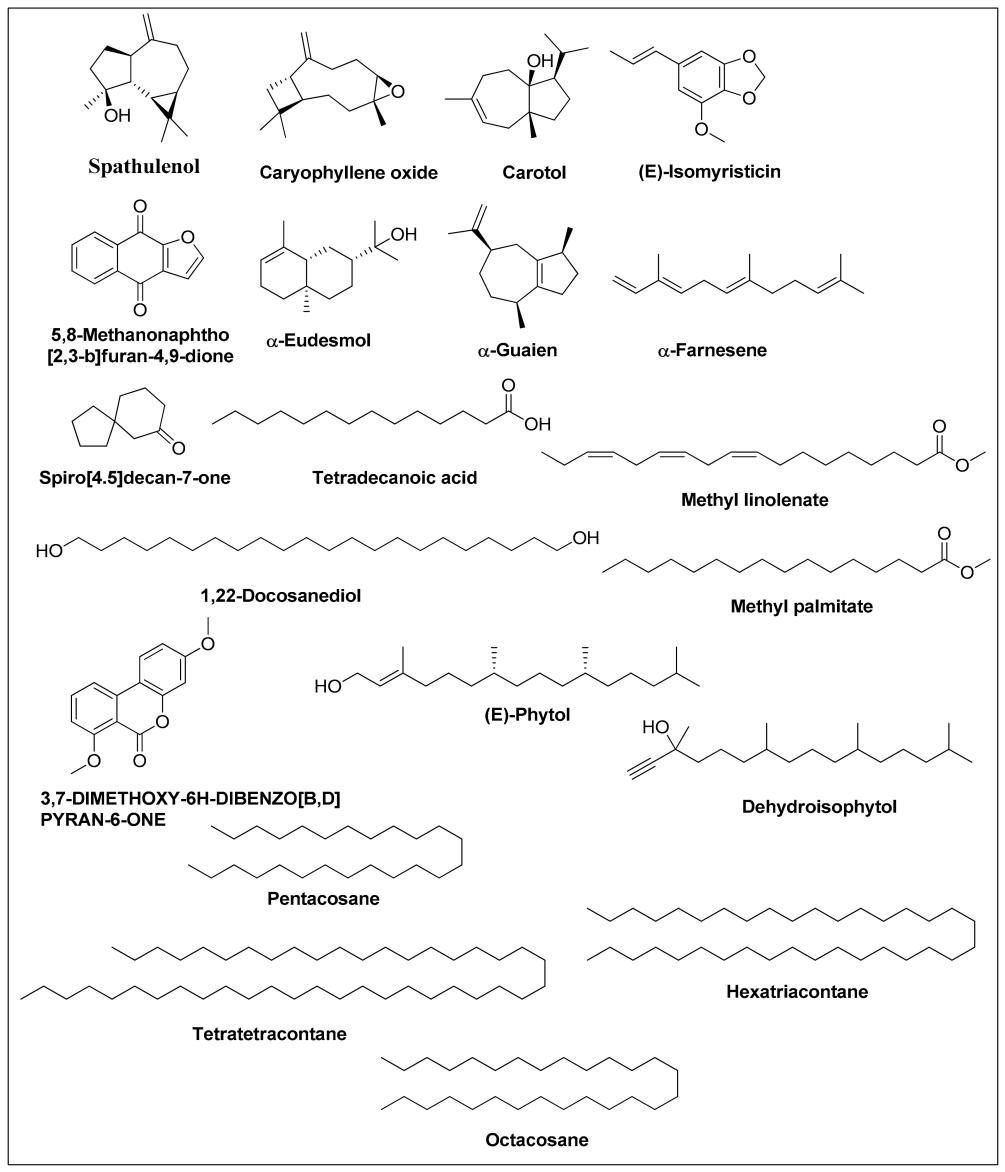
Structures of the identified compounds in *A. orientale* aerial parts essential oil.

A thorough gas chromatography-mass spectrometry (GC-MS) analysis was implemented on the essential oil derived from *A. orientale* (Figure 2). The oil output per dried plant weight was 1.51% (*w*/*w*) of the volume. Sixty components in all were found and measured, making up 99.9% of the total components. Based on their elution sequences, the associated names and structures of these components are shown in Table 1 and Figure 3, respectively. Alloocimene (3.77%), 5,7-dimethylenebicyclo [2.2.2] oct-2-ene (3.15%), camphene (2.94%), and terpinolene (2.87%) were the main identified monoterpene hydrocarbons, while Spiro [4.5] decan-7-one (5.68%) was the main identified oxygenated monoterpene component. The results exhibited that *α*-Cedrene (5.86%), ylangene (4.89%), Bicyclo [7.2.0]undec-3-ene, 4,11,11-trimethyl-8 methylene (1.84%), and *α*-Selinene (1.76%) were the major sesquiterpene hydrocarbons (four constituents representing 14.35%), whereas the main oxygenated sesquiterpenes (two ingredients representing 3.48%) were isolongifolan-8-ol (1.71%) and caryophyllene oxide (1.77%). Non-terpene derivatives and other relevant hydrocarbons account for 26.26 percent.

Plant metabolites have remarkable properties, such as anticancer and other significant biological functions. A broad line of data has demonstrated that EOs, one of the important secondary plant metabolites, exhibit various biological actions. [18,19]. In terms of chemistry, EOs come from the isoprenoid pathways and are a complex mixture of hydrocarbons and oxygen-rich hydrocarbons that are biosynthesized. Monoterpenes and sesquiterpenes make up the majority of these compounds [20]. Following GC-MS analysis of the EO extracted from *A. orientale*, 60 components totaling 99.9% of the components were identified and quantified; these comprised sesquiterpene hydrocarbons, oxygenated sesquiterpenes, monoterpene hydrocarbons, and oxygenated monoterpenes. Refer to (Figure 2 and Figure 3) and (Table 1).

Prior research on the EO extracted from the aerial parts of *A. orientale* by hydro-distillation revealed that it was abundant in monoterpene compounds. The study found that limonene, α-terpinolene, and myristicin were the main oil constituents of the aerial parts [21]. In a different investigation, the essential oil of *A. orientale*, as determined by GC-flame ionization detector and GC-MS, was rich in monoterpene hydrocarbons, including phenylpropanoid myristicin, limonene, and myrcene, and the sesquiterpene compound, germacrene D [22]. This was consistent with our findings, which indicated that myristicin, isomyristicin, and terpinolene were among the main constituents of EO, albeit in varying amounts. The constituents found by a study conducted by [21] on the EO of *A. orientale*, such as *α*-pinene, *β*-pinene, *p*-cymene, and *β*-caryophyllene, were consistent with our analysis for the same compounds. Both our study and the EO composition of *Anisosciadium lanatum* Boiss have reported the presence of camphene, caryophyllene oxide, *α*-selinene, *α*-phellandrene, carene, *α*-eudesmol, isoeugenol, *α*-humulene, *β*-farnesene, terpinolene, *p*-cymene, *β*-caryophyllene, *α*-pinene, *β*-pinene, and *α*-guaien [23]. According to our study, myristicin, 5,8-methanonaphtho [2,3-b]furan-4,9-dione, isomyristicin, *α*-Cedrene, 7-methylenebicyclo[4.2.0]oct-2-ene, spiro[4.5]decan-7-one, ylangene, 5,7-dimethylenebicyclo[2.2.2]oct-2-ene, alloocimene, terpinolene, camphene, and tetratetracontane were the most prevalent compounds in the *A. orientale* EO composition. It is worth noting that the chemical constituents of EOs may be impacted by several factors, including the plant’s location, harvest season, climate, soil type, age of the plant parts, the state of the utilized plant materials, the portion of the plant used, the collection date, and the chemotype [24,25].

### 2.2. In Vitro Antioxidant Activities

#### 2.2.1. Hydrogen Peroxide Scavenging Activity of *A. orientale* EO

Reactive oxygen species (ROS) encompass a diverse array of compounds and free radicals that are derived from molecular oxygen [26]. Examples of ROS comprise free radicals, for instance, superoxide anion radicals (O_2_^−^) and hydroxyl radicals (OH), as well as non-free radical species as hydrogen peroxide (H_2_O_2_) and singlet oxygen (1O_2_) [27]. Free radicals may originate from both internal and external sources. Endogenous factors can be, for example, inflammation and cancer [28]. H_2_O_2_ is widely recognized as a cytotoxic agent, necessitating reduction by antioxidant defense enzymes [29].

**Figure 4:**
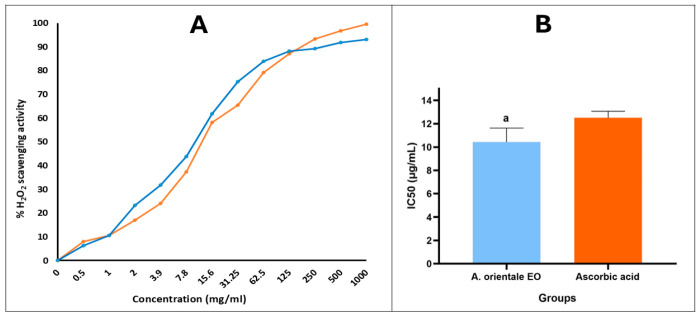
Dose-dependent (**A**) and IC_50_ (**B**) of the H_2_O_2_ scavenging activity of the *A. orientale* EO and ascorbic acid. The data, obtained from three replicates, are stated as mean ± SD, as (^a^) *p* < 0.05, related to the ascorbic acid group. The sample (blue) and the standard (orange).

The results of the current study displayed that the scavenging effect of *A*. *orientale* EO is significantly less than that of ascorbic acid by 17% (Table 2 and Figure 4), as indicated by the dosage-dependent linear standard curve of H_2_O_2_-scavenging activity. Along with the free radical scavenger activity of myricetin, the presence of phenolic compounds, including camphene, as well as triterpenes, may account for this potent H_2_O_2_-scavenging activity of the EO. Phenolic chemicals are acclaimed for their potent antioxidant properties, including their ability to eliminate hydrogen peroxide (H_2_O_2_), a reactive oxygen species that induces oxidative stress and cellular damage [30]. In vitro studies indicate that extracts rich in phenolic compounds from various plants can effectively scavenge H_2_O_2_, often surpassing conventional antioxidants such as ascorbic acid. This effect is closely associated with the total phenolic content of the extract, highlighting the significance of these compounds in counteracting free radicals and safeguarding against oxidative damage [31]. Although it is not a phenolic component by itself, camphene is a bicyclic monoterpene that is commonly noticed in essential oils that include phenolics and have potent antioxidant properties [32]. Studies have demonstrated the effectiveness of camphene and its derivatives in eliminating free radicals, including the neutralization of H_2_O_2_ and other reactive oxygen species [33,34].

#### 2.2.2. ABTS Radical Scavenging Activity of *A. orientale* EO

**Figure 5:**
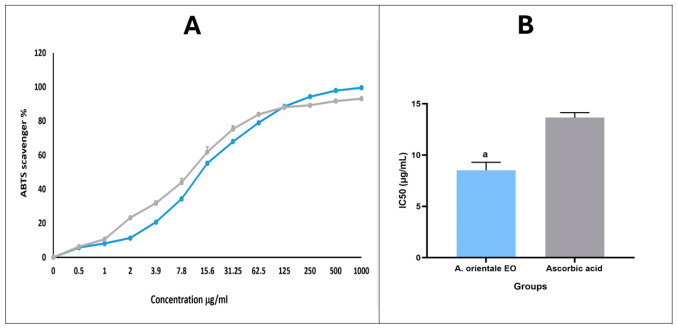
Dose-dependent curve (**A**) and IC_50_ (**B**) of the ABTS scavenging activity of the *A. orientale* EO and ascorbic acid. The data, obtained from three replicates, are stated as mean ± SD, as (^a^) *p* < 0.05, related to the ascorbic acid group. The sample (blue) and the standard (grey).

*A. orientale* EO’s capacity to capture the ABTS free radical was used to estimate its antioxidant activity. (Figure 5) demonstrates the test outcomes for calculating the IC_50_ and the proportion of inhibition of the ABTS radical scavenging activity. The IC_50_ value of a phytochemical molecule indicates its antioxidant capacity, as it represents the amount of antioxidant content required to decrease the concentration of free radicals by 50%. The results (Table 2) imply that the EO exhibited a similar level of free radical inhibition to that of ascorbic acid across all concentrations tested. In addition, the EO induced a lower IC_50_ value of 8.51± 0.79 than the IC_50_ value of ascorbic acid, 13.65 ± 0.48.

Although monoterpene hydrocarbons have been found as the primary component of the oil, myristicin, the main source of phenolics in the essential oil, has been identified as the most abundant element in *A. orientale* EO. Multiple studies have documented myricetin’s antioxidant and free radical-scavenging capacity. In vitro models using DPPH and ABTS assays have shown that myricetin exhibits free radical scavenging activity against several ROS, including hydroxyl radicals, hydrogen peroxide, and superoxide anions [35]. Because of its hydroxyl group-rich composition, it can transfer electrons or hydrogen atoms, stabilize free radicals, and avoid cellular injury [36]. Additionally, even at low levels, myricetin prevents oxidative damage to cells and diminishes intracellular ROS levels in cell-based models [35].

### 2.3. Cytotoxic Activity of A. orientale EO

Cancer ranks among one of the foremost causes of mortality worldwide. Several complications were linked to the chemotherapeutic agents, including drug resistance or a lack of selectivity for cancer cells. These problems necessitate further study and the development of effective remedies [37]. The development of innovative anticancer therapies may benefit from the use of natural chemicals obtained from medicinal plants [38]. To reverse the stages of carcinogenesis, a natural compound capable of triggering apoptosis and growth arrest in cancer cells while sparing healthy cells from cytotoxicity is necessary [39].

**Figure 6:**
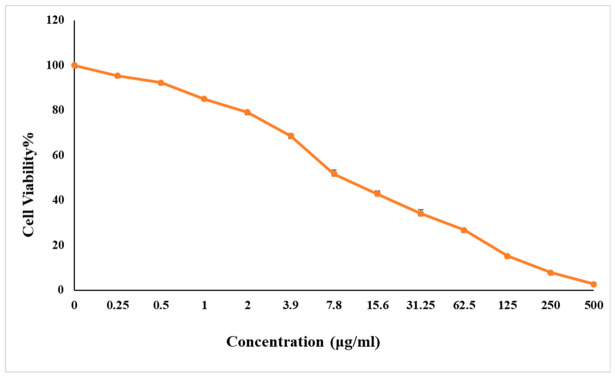
Dose-dependent viability of A549 against the MRC-5 cell line after treatment with *A. orientale* EO.

The antiproliferative effects of *A. orientale* EO, as well as doxorubicin, on human cancer cell lines (HepG2, A549, and HL-60) against the normal human lung fibroblast cells (MRC-5) were assessed using a standard MTT assay protocol as depicted in (Figure 6). The cells were subjected to different concentrations (0.25 to 500 μg/mL) of *A. orientale* EO, or doxorubicin. The percentage of surviving cells was determined relative to the untreated cells.

The IC_50_ values for *A. orientale* EO and doxorubicin against the four human cell lines are shown in Table 3. The IC_50_ values for *A. orientale* EO were 9.31, 99.0, 84.8, and >100 μg/mL for MRC-5, HepG2, A549, and HL-60, respectively. The IC_50_ values for doxorubicin were 26.1, 21.6, 28.3, and 45.6 μg/mL for the same cell lines. These anticancer effects of *A. orientale* EO can be attributed primarily to its content of myristicin.

Along with our results, myristicin was reported to be the active anticancer and antioxidant agent in the oils of several plants, including *H. pastinacifolium* [40], seeds of *Myristica fragrans* [41], parsley seed [42] and parsley (*Petroselinum crispum*) essential oil [43]. Additionally, one study showed that the cytotoxic effects of EO and extracts of *A. orientale* on three human cancer cell lines (HeLa, LS180, and Raji) were attributed to its content of myristicin [22].

Moreover, the observed potent antitumor effects of the EO may also be attributed to its content of camphene. As documented by several cellular and animal studies, camphene is a potent anticancer agent operating via stimulation of apoptosis in cancer cells, inhibition of tumor proliferation, and prevention of cellular division [44,45,46].

### 2.4. Apoptotic Activity

**Figure 7:**
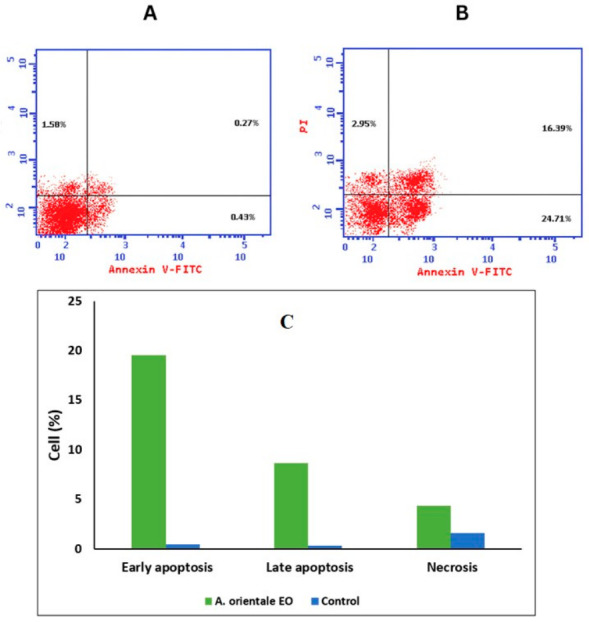
Images from the Annexin V/PI apoptosis test demonstrate how *A. orientale* EO causes apoptosis in A549 cancer cells after treatment at an IC_50_ concentration for 72 h. (**A**) represents untreated A549 cells. (**B**) represents treated A549 cells. (**C**) Apoptotic effects of *A. orientale* EO on lung cancer cell line (A549).

The proapoptotic efficacy of the *A. orientale* EO was assessed in A549 cells at the IC_50_ concentration (84.8 µg/mL) for 72 h, using an Annexin V/Propidium Iodide assay. (Table 4 and Figure 7) illustrate that treatment of the cell lines with *A. orientale* EO exhibited a significant rise in the proportion of Annexin V-positive cells, reflecting a considerable elevation in the number of apoptotic cells. A549-treated cells demonstrated early and late apoptotic rates of 19.53% and 8.66%, respectively, whereas control cells displayed rates of 0.43% and 0.27%.

To determine the possible mechanism underlying the decreased cancer cell viability following EO administration, the study investigated apoptotic markers and lung cancer cell-cycle arrest. Early and late-stage apoptotic cells exhibited alterations, such as the absence of phospholipid asymmetry [47].

**Figure 8:**
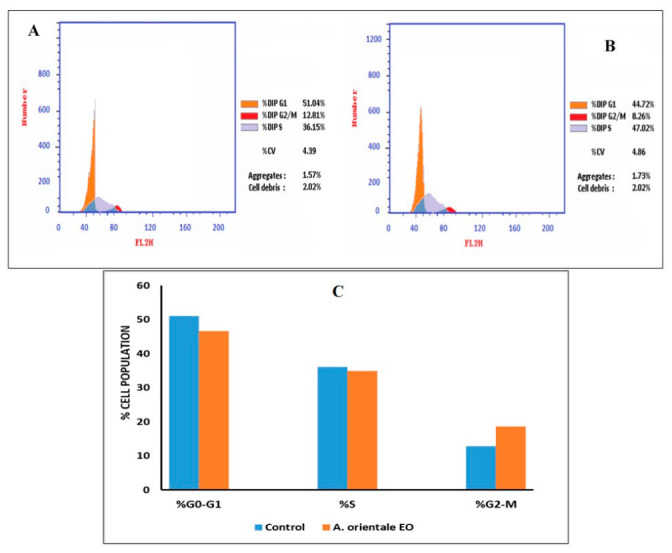
DNA content analysis utilizing flow cytometry was accomplished to evaluate the effect of *A. orientale* EO on A549 cancer cells after 72 h of therapy at IC_50_ concentration (84.8 μg/mL). (**A**) represents the cell cycle histogram of untreated A549 cells. (**B**) represents the cell cycle histogram of treated A549 cells. (**C**) Effect of *A. orientale* EO on A549 cell cycle.

Flow cytometric analysis was performed to investigate the effect of *A. orientale* EO on cell-cycle distribution in A549 cells. Treatment with *A. orientale* EO at its IC_50_ concentration (84.8 μg/mL) altered the distribution of cells across the cell-cycle phases compared with untreated control cells. The percentage of cells in the G2/M phase increased from 12.81% in control cells to 18.62% in treated cells. Concurrently, the proportion of cells in the G0/G1 phase decreased from 51.04% to 46.53%, while the S phase population slightly decreased from 36.15% to 34.85%. These findings suggest an accumulation of cells in the G2/M phase following treatment with *A. orientale* EO, indicating a potential interference with cell-cycle progression at this checkpoint (Table 5; Figure 8).

The rise in the number of cells in the G2/M phase indicates a higher rate of cell death due to DNA damage and biochemical pathways involving signaling cascades [48]. Many naturally occurring phytochemicals are believed to disrupt the cell cycle and promote apoptosis, thereby inhibiting cancer cell growth [49]. Cancer proliferation is primarily regulated by the cell cycle [50,51], which encompasses four distinct, sequential phases: G1, sub-G1, S, and G2/M [52].

### 2.5. Molecular Mechanisms of A. orientale EO Apoptotic Activity

**Figure 9:**
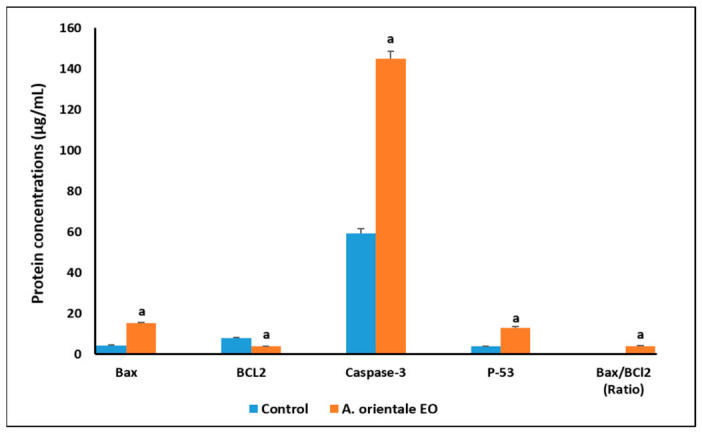
Effect of *A. orientale* EO on apoptotic protein levels (Bax, BCL2, caspase 3, and p53, as well as in Bax/Bcl2 ratios). The data, obtained from three replicates, are stated as the mean ± SD. (^a^) *p* < 0.05, related to the positive control.

Apoptosis and anti-apoptosis intracellular processes were investigated to understand the molecular mechanisms behind the activation of apoptosis and G2/M phase block. Comparing the test group to the control, *A. orientale* EO treatment markedly raised the levels of various apoptotic indicators. The levels of Bax and Caspase-3 increased by 263.25% and 144.07%, respectively (Table 6 and Figure 9). Additionally, there was an enhanced Bax/Bcl2 ratio of 643.89% and a 229.41% increase in p53 levels. In the meantime, the treated group’s Bcl-2 level significantly decreased by 51.03%.

These observed changes indicate that the treatment predominantly triggers apoptosis, as demonstrated by the upregulation of proapoptotic factors expression and downregulation of anti-apoptotic protein Bcl-2 expression. The expression levels of Bcl-2 family members, characterized by low anti-apoptotic Bcl-2 and elevated proapoptotic Bax and p53, alongside caspase cascade activity, indicate the crucial role of Bcl-2 and caspase pathways in the induction of apoptosis of the A549 lung cancer cell line.

P53, or tumor protein p53, is an essential tumor suppressor protein that is pivotal in inhibiting cancer development [53]. p53 expression is typically low and exists as a functionally latent protein. The p53 signaling pathway is activated in response to various stress signals due to DNA damage, allowing cells to initiate multiple transcriptional programs such as senescence, cell-cycle arrest, DNA repair, and apoptosis, inhibiting tumor growth [54].

The findings regarding pro- and anti-apoptotic protein levels indicate the contribution of the intrinsic pathway in cell death and imply that apoptosis triggered by *A. orientale* EO may be p53-dependent, as evidenced by the alterations in Bcl-2 and Bax expression associated with p53-dependent apoptosis. Most human malignancies are associated with the loss of function or negative regulation of p53 proteins, resulting in the inactivation of the Tp53 gene [55]. The stimulation of p53-dependent apoptosis by *A. orientale* EO shows potential for the treatment of this specific type of cancer. Numerous studies reported that a majority of cancer cells possess mutant or deleted p53; thus, the efficacy of in vivo apoptosis stimulation may be contingent upon a comprehensive examination of pro- and anti-apoptotic proteins [53,56,57]. Following treatment of A549 cells with *A. orientale* EO, ELISA demonstrated a significant increase in p53, caspase-3, and Bax, alongside a reduction in Bcl-2 protein levels. These results imply that *A. orientale* EO may act as a potential therapeutic agent for lung carcinoma.

Interestingly, the cytotoxic activity of *A. orientale* EO differed among the tested cancer cell lines, with HL-60 cells showing the lowest sensitivity (IC_50_ > 100 μg/mL). This finding may be related to the p53 status of the cell line. HL-60 cells are known to be deficient in functional p53 due to TP53 deletion, whereas p53 signaling plays a critical role in regulating cell-cycle arrest and apoptosis.

Several research papers have shown that p53 plays a dual role in apoptosis by directly and indirectly modifying Bcl-2 family proteins, inhibiting cell development, and activating apoptotic pathways that lead to programmed cell death [55,58,59]. It has been proposed that either functionally inhibiting anti-apoptotic Bcl-2 family members or overexpressing their proapoptotic counterparts may restore the apoptotic machinery in tumor cells and enhance their sensitivity to chemotherapy and radiotherapy [60]. Our investigation showed that *A. orientale* EO elevated the Bax/Bcl2 ratio and induced p53 overexpression in A549 cells following treatment.

Due to its ability to restore apoptotic pathways and increase tumor susceptibility to radiation and chemotherapy, *A. orientale* EO is positioned as a prospective chemopreventive drug for various tumors. The caspase cascade plays a critical role in initiating apoptosis via intrinsic or extrinsic pathways. The intrinsic and extrinsic apoptotic signaling pathways are closely associated with caspase-3 enzyme activity in treated cells. Caspase-3 activation, induced by caspase-9, subsequently leads to the activation of caspase-8, which is the final step in the process of cell death [61]. The cellular genome undergoes genomic aberrations due to mutation, deletion, and/or translocation, resulting in genomic instability. Cancer often develops due to the accumulation of genetic abnormalities. A proper stress response is essential for preserving genomic integrity and protecting cells from malignant transformation [62].

Since our results demonstrated a marked increase in p53 expression following treatment of A549 cells with *A. orientale* EO, together with increased Bax and caspase-3 levels and reduced Bcl-2 expression, the reduced responsiveness of HL-60 cells may suggest that the cytotoxic effects of the essential oil are mediated, at least in part, through a p53-dependent apoptotic pathway. Further studies are required to confirm the precise molecular mechanisms underlying the differential sensitivity of these cell lines.

### 2.6. EGFR Inhibition Effect of A. orientale EO

**Figure 10:**
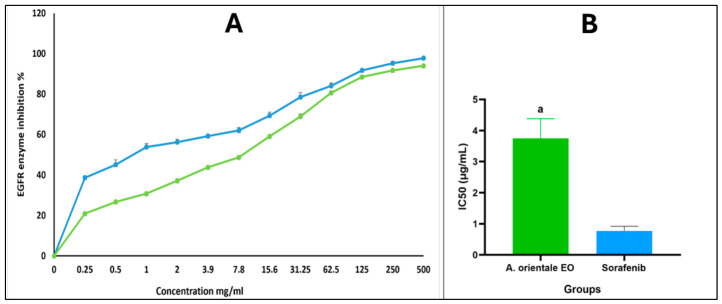
Dose-dependent curve (**A**) and IC_50_ (**B**) of the EGFR enzyme inhibition of *A. orientale* EO and *sorafenib*. The data, obtained from three replicates, are expressed as mean ± SD, as (^a^) *p* < 0.05, related to the ascorbic acid group. The sample (green) and the reference standard (Blue).

Epidermal growth factor receptor (EGFR) inhibition has significant effects on cellular proliferation. In the current study, treatment with *A. orientale* EO demonstrated a dose-dependent inhibition of A549 cell line proliferation related to the EGFR inhibitor sorafenib, which was used as a positive control. Growth factors are molecules that resemble hormones and can encourage normal cell migration and division. However, cancer has figured out the underlying mechanisms that allow metastasis and tumor growth. EGFR is the original member of growth factor receptors with intrinsic tyrosine kinase activity. The overexpression of EGFR correlates with several cancer types [63,64]. Consequently, targeting EGFR with small-molecule inhibitors is an established and effective technique in cancer treatment [65]. These results showed that *A. orientale* EO displayed dose-dependent inhibition of cell growth in A549 cell lines compared to the EGFR inhibitor sorafenib (Table 7 and Figure 10). This inhibition may be due to the myristicin content in EO, which was supported by the highest docking score with EGFR TK. In line with our results, myristicin has been shown to have a potent anticancer effect on gastric cancer cells via inhibiting the EGFR/ERK signaling pathway. It might be used as a novel medication to treat stomach cancer [66].

### 2.7. Molecular Docking Studies

The epidermal growth factor receptor tyrosine kinases family has been identified as one of the most distinctive targets of cancer [67]. Many human malignancies, including lungs, glioblastoma, and epithelial tumors of the head and neck, are linked to the overexpression of EGFR kinase, which has prompted the creation of anticancer treatments that target EGFR [68]. By using the co-crystallized ligand (W19b) as a reference molecule, the binding interactions between the studied phytochemical ingredients and the EGFR enzyme could be interpreted in this work.

#### 2.7.1. Molecular Docking Analysis

During the CDOCKER simulation, 10 binding poses were generated for each ligand within the defined active site. The generated poses were evaluated based on their CDOCKER energy and CDOCKER interaction energy values, and the most favorable conformation was selected for further study. These C-DOCKER scores are expressed as negative values; therefore, more negative scores indicate stronger and more favorable binding interactions between the ligand and the target protein. The docking poses were ranked according to their CDOCKER energy and CDOCKER interaction energy values, and the highest-ranked conformation was selected for interaction analysis. No formal clustering analysis of the generated poses was performed.

**Figure 11:**
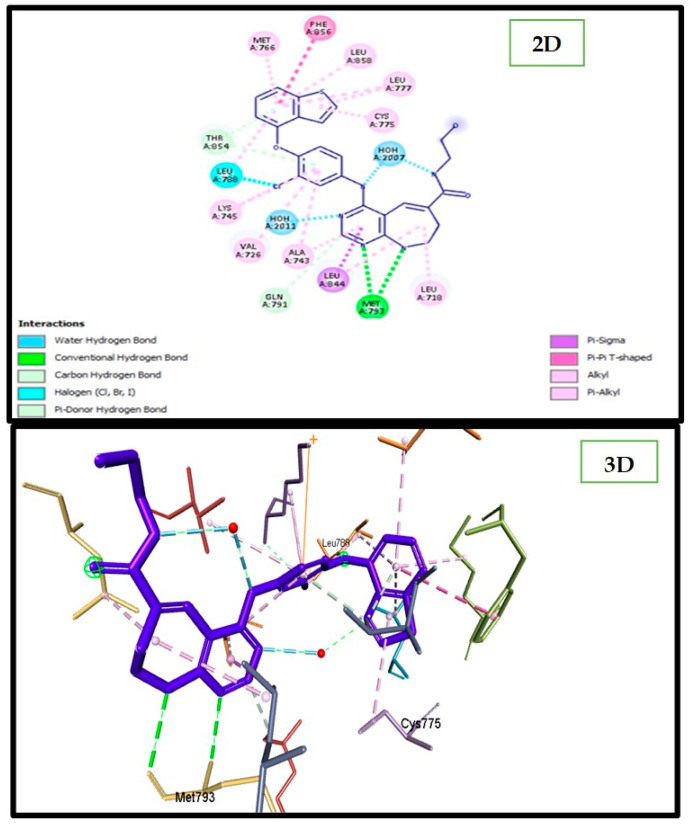
2D and 3D molecular interactions between EGFR residues and W19.

The co-crystallized ligand (W19) exhibited a characteristic binding mode involving a key hydrogen bond with the hinge region residue MET793, which is a critical interaction responsible for anchoring ATP-competitive EGFR inhibitors. This interaction is considered one of the hallmark features of EGFR inhibition, as hydrogen bonding with hinge-region residues contributes significantly to ligand recognition, binding stability, and kinase selectivity. Similar observations have been reported in previous molecular docking and structure-based drug design studies of EGFR inhibitors [69,70]. In addition, multiple hydrophobic contacts with residues such as CYS797, LEU718, ALA743, LEU777, LYS745, and VAL726 further stabilized the ligand within the active site, consistent with previously reported EGFR inhibitory scaffolds, (Figure 11). Redocking of (W19) reproduced the experimental binding pose with an RMSD of 1.38 Å, confirming the reliability of the CDOCKER protocol for predicting accurate binding orientations in the EGFR active site.

The goal of the docked complex analysis was to provide a qualitative evaluation of the molecular underpinnings of the biological activity of closely linked molecules. Considering that assays have been conducted in vitro, which increases the difficulty of the biological response and, consequently, of connection to theoretical data, this was used to draw a primary structure activity correlation.

**Figure 12:**
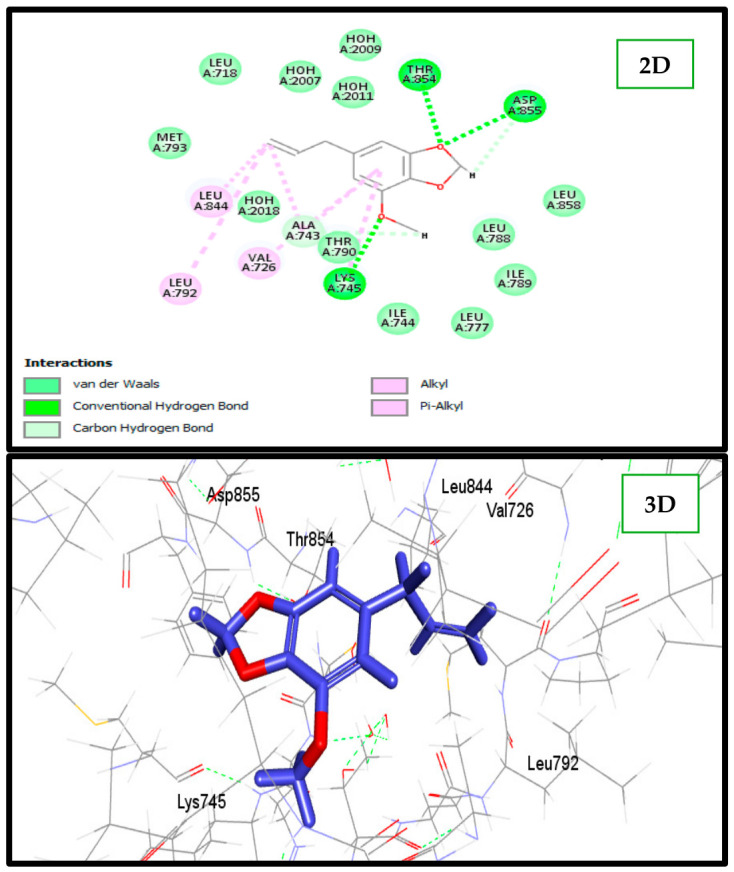
2D and 3D molecular interactions and amino acids involved between Myristicin and the EGFR active site.

Among the tested compounds, myristicin, (Z)-isomyristicin, and bicyclo[7.2.0]undec-3-ene derivatives exhibited the most favorable CDOCKER interaction energies (−33.36 to −32.61 kcal/mol). These compounds demonstrated conserved interactions within the ATP-binding pocket, including hydrogen bonding with ASP855 and THR854, alongside extensive hydrophobic stabilization mediated by residues such as LEU844, LEU788, VAL726, and LYS745, (Figure 12). Hydrogen-bond interactions with residues ASP855 and THR854 may contribute to stabilization of the ligand within the ATP-binding cleft and enhance binding specificity. Similar interactions have been reported to strengthen ligand accommodation and improve binding affinity in EGFR-targeted molecular docking studies [69]. Although these ligands did not fully replicate the hinge-binding interaction observed with (W19), their ability to occupy the hydrophobic cavity suggests a potential competitive binding mode.

The docking results for the phytochemical constituents that were recognized by GC-MS in *A. orientale* EO against the active site of the EGFR Tyrosine Kinase Domain are listed in (Table 8) which depicts the binding scores and details of the binding relations, including their conforming bond categories, for the phytochemical compounds as well as the co-crystallized ligand (W19).

The observed differences in docking scores among the phytochemical constituents can be rationalized based on structural features. In particular, oxygenated aromatic systems such as myristicin and isomyristicin provide enhanced polar interaction capability, which likely contributes to improved binding affinity compared to purely hydrocarbon-based terpenoids, which mainly rely on hydrophobic contacts. This structure–activity relationship suggests that the presence of polar functional groups improves binding orientation stability within the EGFR active site.

Overall, the docking analysis indicates that the identified phytochemical constituents can accommodate the EGFR binding pocket and engage key catalytic site residues, although with varying degrees of interaction strength. The dominance of hydrophobic interactions reflects the lipophilic nature of the binding cavity, while hydrogen bonding with residues such as ASP855 and THR854 contributes to binding specificity. These findings support the potential of the tested compounds as moderate EGFR binders and provide a structural basis for their observed in vitro activity.

#### 2.7.2. Validation of the Docking Protocol

The goal of the docking protocol is to anticipate the ligand’s pose, or orientation and conformation, in relation to the protein’s binding site. Therefore, distinguishing between recommended poses, which are typically explained as poses that are within 2.0 Å root mean square deviation (RMSD) from X-ray geometry, and false or mis-docked poses is the main objective of a good docking process.

**Figure 13:**
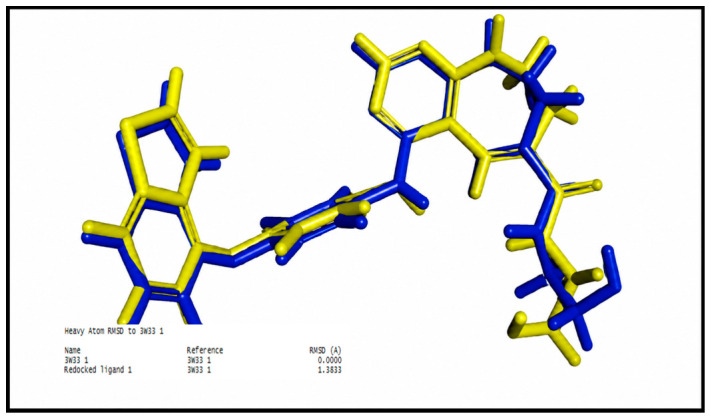
The alignment among the redocked (W19) posture obtained by docking using CDOCKER (blue) and the co-crystallized bioactive conformer of the parent ligand (W19) (yellow).

We rebuilt the co-crystallized ligand (W19) using C-DOCKER and aligned the pose recovered from docking to the X-ray geometry (the co-crystallized bioactive conformation) to compute the RMSD in order to verify the predictability of the proper poses using the C-DOCKER procedure. The CDOCKER technique is valid to estimate the pose of the inhibitors in the X-ray model of the EGFR active site because the docking of (W19) in EGFR (PDB: 3W33) produced an RMSD of 1.38 Å (Figure 13).

## 3. Materials and Methods

### 3.1. Chemicals and Reagents

Bax (kit ref. Bax, Catalog No. MBS935667, MyBioSource, Inc., San Diego, CA, USA; sensitivity, 15.6 pg/mL), Bcl-2 (kit ref. Bcl-2, Catalog No. BMS244-3, Invitrogen, Inc., San Diego, CA, USA; sensitivity, 0.5 ng/mL), p53 (kit ref. rat p53 Catalog. No. MBS355295, MyBioSource, Inc., San Diego, CA, USA; sensitivity, 10 pg/mL), and caspase-3 (kit ref. caspase-3, Catalog No. MBS261814, MyBioSource, Inc., San Diego, CA, USA; sensitivity, 0.5 ng/mL). EGFR Kinase Assay Kit (BPS, Bioscience, San Diego, CA, USA). MTT, Dimethyl sulfoxide (DMSO), and trypan blue dye were bought from Sigma (St. Louis, MO, USA). Fetal Bovine serum, RPMI-1640, HEPES buffer solution, 0.25% Trypsin-EDTA, gentamycin, and L-glutamine were gotten from Lonza, Antwerp (Belgium), and anhydrous sodium sulfate (Lanxess, Thane, India).

### 3.2. Plant Collection and Distillation of Essential Oil

*Anisosciadium orientale* aerial parts were gathered from Hail, Saudi Arabia (29°23′14.8″ N 43°30′05.2″ E) (27°56′31.9″ N 41°49′56.9″ E) in spring (8 April 2024). Dr. Naila Hassan Alkefai, University of Hafr Al-Batin, identified the plant and verified the name using https://www.worldfloraonline.org/taxon/wfo-0000537391 (accessed on 19 April 2024). A voucher specimen with the number UOHCOP016 was placed at the Pharmaceutical Chemistry Department, University of Hail. The aerial parts of *A. orientale* were blended into a powder and hydro-distilled for three hours at a boiling temperature of around 100 °C under air pressure in a Clevenger-style device. After that, the extracted essential oil was gathered and dried on anhydrous sodium sulfate. The obtained yield was 1.51% (*w*/*w*). Until additional analysis, the oil samples were saved in dark glass containers at 4 °C [2].

### 3.3. GC/MS Essential Oil Analysis

Utilizing a Thermo Trace 1300 gas chromatograph (Thermo Fisher Scientific, Rodano, Milan, Italy) fitted with an AI 1310 autosampler (Thermo Fisher Scientific, Rodano, Milan, Italy) and connected to a Thermo ISQ 7000 mass spectrophotometer (Thermo Fisher Scientific, Rodano, Milan, Italy), the samples were dissolved in hexane and subjected to GC/MS analysis. For separation, a merged silica capillary column with a DB-5 stationary phase (0.32 mm in ID, 0.25 mm in thickness, and 30 m in length) was employed. At a flow rate of 1 mL/min and a pressure of 13 psi, helium was applied as the carrier gas. With an ion foundation temperature of 250 °C, an MS transfer temperature of 290 °C, an ionization voltage of 70 eV, a *m*/*z* variety of 40–800 amu, and an injection capacity of 1 µL with spitless mode, the temperature programming involved an early temperature of 60 °C held for 5 min, afterward increased to 280 °C at a proportion of 5 °C/min and held at 280 °C for another five minutes. The comparative retention durations and mass spectra were matched with those in Wiley and NIST databases in order to recognize the components.

### 3.4. In Vitro Antioxidant Assessment

#### 3.4.1. In Vitro Evaluation of the Scavenging Activity of Hydrogen Peroxide (H_2_O_2_)

The antioxidant activity of *A. orientale* essential oil was estimated at the Regional Center for Mycology and Biotechnology at Al-Azhar University, Cairo, Egypt, by the ABTS (2,2′-azino-bis (3-ethylbenzothiazoline-6-sulfonic acid); free radical scavenging and hydrogen peroxide (H_2_O_2_) radical scavenging were examined in triplicate, and mean values were obtained. A solution of H_2_O_2_ (43 mM^−1^) was set in phosphate buffer, pH 7.4. A portion of 0.2 mL sample in distilled water (using different dilutions) was added to the H_2_O_2_ liquid (600 µL of 43 mM^−1^). After ten minutes, the intensity of absorption of H_2_O_2_ at 230 nm was measured and compared to a phosphate buffer blank solution devoid of H_2_O_2_ [71]. The H_2_O_2_ radical scavenging percentage of the samples was determined using the following formula:

H_2_O_2_ radical scavenging % = [(A _blank_ − A _sample_)/A _blank_] × 100.

GraphPad Prism software (Version 8.0.2, San Diego, CA, USA) was used to design the dose–response curve and predict the 50% inhibitory concentration (IC_50_), which is the concentration needed for 50% radical scavenging activity.

#### 3.4.2. In Vitro Assessment of the ABTS Radical Scavenging Activity

The ABTS radical scavenging test is a spectrophotometric decolorization method utilized to quantify the antioxidant capacity of test samples, according to their capacity to neutralize ABTS radicals generated through chemical oxidation [72]. Antioxidant activity was determined using the ABTS radical scavenging test, following the procedure outlined by Samaniego Sánchez and Ling [73,74]. The ABTS radical cation (ABTS^+^) was made by reacting a 1.8 mM of ABTS stock solution (Sigma, PN: A3219) with 0.63 mM of potassium persulfate. After that, it was incubated for 12 to 16 h at room temperature in the dark before use. An absorbance of 0.700 at 734 nm was then obtained by diluting the resultant solution with ethanol. All spectrophotometric measurements were conducted at room temperature. The test samples were set by dilution with 80% methanol at a 1:10 ratio. Subsequently, 190 μL of the ABTS radical solution was combined with 10 μL of the diluted samples in a microtiter plate. The amount of absorption at 734 nm was recorded at 1-min intervals for up to 13 min following the initial stirring. To guarantee accuracy, solvent blanks were used in every test. Ascorbic acid served as the reference antioxidant, while methanol was set as the negative control. Each experiment was carried out in triplicate, with three separate copies of each sample. This formula was used to estimate the proportion of free radical scavenging activity.

% Free Radical Scavenging Activity = [(An − As) × 100]/An

where (An) is the last absorbance value of the negative control, and (As) represents the last absorbance value of the sample.

### 3.5. Assessment of Cytotoxicity in Vitro

#### 3.5.1. Cell Lines and Culture Medium

Mammalian cell lines: MRC-5 cells (Normal human Lung fibroblast), HePG 2 (Human hepatocellular carcinoma), A549 (Lung carcinoma), and HL-60 (Human Leukemia-60) were acquired from the American Type Culture Collection (ATCC, Rockville, MD, USA).

Cell line Propagation: Cells were cultured in RPMI-1640 medium accompanied with 10% heat-inactivated fetal calf serum in addition to 50 μg/mL gentamycin. Cultures were retained at 37 °C in a wet incubator with 5% CO_2_ and sub-refined two to three times per week to maintain exponential growth.

#### 3.5.2. Cytotoxicity Evaluation Using Viability Assay

For the cytotoxicity assay, cells were broadcast into Corning^®^ 96-well tissue culture plates at a density of 1 × 10^4^ cells per well in 100 μL of growth medium and incubated for 24 h. The essential oil of *A. orientale* was subsequently applied in twelve concentrations, with three replicates per concentration. Each plate included six vehicle controls containing either culture medium or 0.5% DMSO. Following a 72-h incubation, cell viability was judged using the MTT assay. Briefly, the culture medium was reinstated with 100 μL of phenol red-free RPMI-1640 medium, and ten μL of a 12 mM MTT stock solution (made by dissolving 5 mg of MTT in 1 mL of PBS) was supplemented to each well, including untreated controls. Plates were incubated at 37 °C in 5% CO_2_ for four hours. Subsequently, 85 μL of medium was eliminated from each well, and 50 μL of DMSO was included, mixed carefully, and sat on at 37 °C for 10 min. The optical density was then exact at 590 nm using a microplate reader (SunRise, TECAN, San Jose, CA, USA). The following formula was used to determine the percentage of viability in order to estimate the total quantity of viable cells:

[(OD_t_/OD_c_)] × 100%

where OD_t_ is the optical density of the wells treated with the tested material, and OD_c_ represents the optical density of the untreated cells [75,76]. After each tumor cell line was treated with the test substance, survival curves were created by plotting the association between drug concentration and cell survival. Using GraphPad Prism software program (Version 8.0.2, San Diego, CA, USA), graphic designs of the dose–response curve for separate concentrations were used to determine the cytotoxic concentration (CC_50_), which is the dosage needed to cause toxic effects in 50% of intact cells [77].

#### 3.5.3. Apoptosis Analysis (Annexin V-FITC Assay)

Apoptotic cells were evaluated using the Annexin V-FITC assay. As previously mentioned, *A. orientale* essential oil was applied to A549 lung cancer cells at the IC_50_ level after they had reached confluence. After 72 h of treatment, cells were removed and given two 20-min PBS rinses, then resuspended in kit binding buffer. A total of 100 μL of binding buffer containing 1 μL FITC-Annexin V (Becton Dickinson BD Pharmingen™, Heidelberg, Germany) was added, and the suspension underwent incubation at 4 °C for 40 min. After incubation, cells were removed and resuspended in 150 μL of binding buffer complemented with 1 μL DAPI (1 μg/mL in PBS; Invitrogen, Life Technologies, Darmstadt, Germany) [78]. The BD FACS Calibur flow cytometer (BD Biosciences, San Jose, CA, USA) was then used to examine the cells.

#### 3.5.4. Cell Cycle Analysis Using Flow Cytometry

The Cycle-TEST^TM^ PLUS DNA Reagent Kit (Becton Dickinson Immunocytometry Systems, San Jose, CA, USA) was used for cell cycle analysis to assess the effect of *A. orientale* essential oil on cell cycle dissemination in the A549 cell line. Following the manufacturer’s instructions, propidium iodide was used to dye both EO-treated and untreated A549 cells. The results were then examined using flow cytometry [79]. Cell-Quest software ((version 5.1, Becton Dickinson Immunocytometry Systems, San Jose, CA, USA) was used to compute the cell cycle dispersion.

#### 3.5.5. Determination of Apoptotic Markers

Untreated A549 cell lysates and those exposed to *A. orientale* essential oil at the previously determined IC_50_ concentration were analyzed for Bax, Bcl-2, p53, and caspase-3 expression. The intra-assay and inter-assay coefficients of variation (CV) for every ELISA kit used in this investigation were less than 15%. The manufacturer’s instructions were strictly followed for conducting experiments and computing results. A Hidex Sense ELISA Multimode plate reader (Hidex Oy, Turku, Finland) was used to quantify absorbance at 450 nm [79].

#### 3.5.6. Assay of EGFR Kinase

EGFR kinase activity was measured utilizing a commercial EGFR Kinase Assay Kit (BPS, Bioscience, San Diego, CA, USA). A potent EGFR kinase inhibitor, sorafenib, was employed as a positive control. [80]. The luminescence was recorded using a Hidex Sense ELISA Multimode plate reader (Hidex Oy, Turku, Finland).

### 3.6. Docking Protocol

Using Accelrys^®^’s Discovery Studio 2.5 (2019) software, docking analyses were performed on the most common EO components found by GC-MS analysis. The EGFR enzyme was used to assess and model the isolated compounds in contrast to the natural co-crystallized ligand: 4-[[4-(1-benzothiophen-4-yloxy)-3-chlorophenyl]amino]-*N*-(2-hydroxyethyl)-8,9-dihydro-7H-pyrimido[4,5-b]azepine -6-carboxamide, which is called (W19b) [81].

The EGFR enzyme’s X-ray crystal structure (PDB code: 3W33) was attained from the Protein Data Bank at the Research Collaboration for Structural Bioinformatics website [https://www.rcsb.org/ (accessed on 9 July 2025)] and then uploaded into the Accelrys Discovery Studio 2.5 software.

The protein structure was prepared using the default protein preparation workflow available in the software. The entire enzyme was energy-minimized through multiple stages using the SMART minimization algorithm. The protein was treated as the receptor, and the binding pocket along with the surrounding amino acid residues were identified. The co-crystallized ligand was removed from the active site following the procedure below:

1. The enzyme PDB file was opened in Discovery Studio.
2. All non-essential chains and water molecules were removed.
3. The structure was then processed via Tools → Protein Report and Utilities → Clean Protein, a step used to add hydrogen atoms and repair any missing amino acid side chains.
4. A CHARMM force field was applied using Tools → Simulate Structure → Forcefield → Apply CHARMM Forcefield.
5. Since hydrogen addition can introduce steric clashes, a minimization step was required. Prior to this, positional constraints were applied to preserve the overall 3D protein conformation.
6. Heavy atoms were selected using Edit → Select → Element not equal to hydrogen → Create Heavy Atoms Group.
7. Fixed atom constraints were assigned to the heavy atom group via Tools → Simulate Structure → Create Fixed Atom Constraints.
8. Energy minimization was then performed through Protocols → Simulation → Minimization → Run.
9. The binding site was defined by setting the protein as the receptor using Tools → Define and Edit Binding Site.
10. Finally, the binding site was defined based on the co-crystallized ligand and the surrounding active-site residues using the “Define Sphere from Selection” option in Discovery Studio. A binding sphere was employed to encompass the ATP-binding pocket and the key amino acid residues involved in ligand recognition and binding. The sphere radius was adjusted to ensure complete coverage of the active-site region.

In addition, the ligand structures were initially built using the sketching tools of Accelrys Discovery Studio 2.5. Furthermore, the designed and lead compounds were constructed using ChemDraw version 12 and subsequently imported into Discovery Studio in MOL format. Hydrogen atoms were then added using the Chemistry → Hydrogens → Add function. All ligands were subjected to energy minimization using the Prepare Ligands protocol under the general-purpose module to obtain energetically favorable conformations prior to docking. The ionization states were adjusted to physiological pH (7.4). During ligand preparation, hydrogen atoms were explicitly added, while the generation of additional stereoisomers and tautomers was disabled to maintain the original structural integrity of the designed compounds. The receptor was defined as the whole enzyme. The binding sites were also found and chosen, as were the nearby amino acid residues. After that, the binding pockets’ Ligand structures were generated using the sketching tools of Accelrys Discovery Studio 2.5 and subsequently prepared using the ligand preparation protocol. Hydrogen atoms were added, and the protonation states were adjusted to physiological pH (7.4). No additional stereoisomers or tautomers were generated during ligand preparation to preserve the original structural features of the compounds. Molecular docking was performed using the CDOCKER protocol implemented in Accelrys Discovery Studio 2.5. The default docking parameters were used, and ten docking poses were generated for each ligand. The docking results were evaluated based on CDOCKER interaction energy to identify the most favorable binding mode. A docking report summarizing ligand poses and their corresponding scores was generated following protocol execution. A total of 10 docking poses were generated per ligand using the CDOCKER protocol, and the highest-ranked pose based on CDOCKER interaction energy was selected for further analysis.

The grid box dimensions were adjusted to fully encompass the binding site and surrounding key residues involved in ligand recognition. The docking method was validated by comparing the binding mode of the lead compound obtained before docking with the ten docked poses and selecting the pose with a binding pattern similar to the lead before docking. The next step was aligning the lead’s X-ray bioactive conformation with the best-fitting posture of the compound and calculating the RMSD.

## 4. Conclusions

After a thorough GC-MS investigation of the essential oil gotten from *Anisosciadium orientale* by hydrodistillation, sixty compounds were identified, with myricetin and its isomers being the main constituents. The EO produced a lower IC50 value than ascorbic acid and demonstrated a similar level of free radical inhibition to that of ascorbic acid across all tested concentrations. The EO showed strong antioxidant and anticancer properties against various cell types. It showed potential toxicity in lung cancer (A549) cells and produced notable apoptotic effects with G2/M cell-cycle arrest. In comparison to the corresponding control, *A. orientale* EO significantly prompted cell-cycle arrest during the G2/M phase. Treatment with *A. orientale* EO significantly lowered expression of the anti-apoptotic protein Bcl-2 and enhanced expression of proapoptotic factors (Bax and Caspase-3). Furthermore, compared to the EGFR inhibitor sorafenib, treatment with *A. orientale* EO reduced cell proliferation in A549 cell lines in a dose-dependent way. The structural resemblance of myristicin and isomyristicin of the 1,3-dioxolobenzene of these constituents to the pyrimido[4,5-b]azepine nucleus of the reference compound was linked to their highest docking scores when compared to the other oil constituents. This study provides the path for more research on the in vivo, preclinical, and clinical application of *A. orientale* EO to treat varied cancer diseases.

## Figures and Tables

**Table 1:** Identified compounds of *A. orientale* aerial parts essential oil.

No.	Rt (min)	Compound Name	Molecular Formula	SI	Area %
1	6.00	*α*-Pinene	C_10_H_16_	646	0.75
2	7.15	Mono-butyl itaconate	C_9_H_14_O_4_	725	0.36
3	7.37	*β*-pinene	C_10_H_16_	664	0.43
4	7.76	*α*-Thujene	C_10_H_16_	790	1.15
5	7.91	*α*-phellandrene	C_10_H_16_	814	1.17
6	8.29	*p*-cymene	C_10_H_14_	841	0.31
7	8.43	Camphene	C_10_H_16_	784	2.94
8	8.66	7-Methylenebicyclo[4.2.0]oct-2-ene	C_9_H_12_	740	5.14
9	8.92	Carene	C_10_H_16_	681	0.97
10	10.10	Terpinolene	C_10_H_16_	868	2.87
11	10.17	5,7-Dimethylenebicyclo[2.2.2]oct-2-ene	C_10_H_12_	629	3.15
12	11.22	Alloocimene	C_10_H_16_	784	3.77
13	12.57	Citral	C_10_H_16_O	628	0.30
14	12.80	8-Hydroxy-p-cymene	C_10_H_14_O	731	1.15
15	12.92	2,2′-Dimethylbenzil	C_10_H_14_O_2_	725	0.93
16	17.29	*α*-Terpinyl acetate	C_12_H_20_O_2_	697	0.85
17	17.50	Isoeugenol	C_10_H_12_O_2_	605	0.47
18	18.33	1-nitro-1-phenylcyclohexane	C_12_H_15_NO_2_	748	0.56
19	18.46	Germacrene A	C_15_H_24_	628	0.25
20	18.71	Methyl eugenol	C_11_H_14_O_2_	581	0.55
21	18.88	*β*-Caryophyllene	C_15_H_24_	735	0.43
22	19.05	Dehydrodihydroionone	C_13_H_20_O	645	0.27
23	19.25	Ylangene	C_15_H_24_	661	4.89
24	19.59	*α*-Cedrene	C_15_H_24_	732	5.86
25	19.79	Benzyl carbinol	C_18_H_10_O	655	0.73
26	20.01	*β*-Farnesene	C_15_H_24_	850	0.25
27	20.11	*α*-Humulene	C_15_H_24_	726	1.22
28	20.65	*β*-Himachalene	C_15_H_24_	779	1.27
29	20.72	*α*-Curcumene	C_15_H_22_	915	0.50
30	20.91	Bicyclo[7.2.0]undec-3-ene, 4,11,11-trimethyl-8 methylene-	C_15_H_24_	713	1.84
31	20.97	Isolongifolan-8-ol	C_15_H_26_O	561	1.71
32	21.12	*α*-Selinene	C_15_H_24_	661	1.76
33	21.20	*α*-Himachalene	C_15_H_24_	600	0.30
34	21.35	*α*-Muurolene	C_15_H_24_	731	0.96
35	21.73	Myristicin	C_11_H_12_O_3_	784	9.43
36	22.29	4-Methyl-2-(2-methyltetrahydrofuran-2-yl)phenol	C_12_H_16_O_2_	654	0.25
37	22.38	*β*-Guajene	C_15_H_24_	637	0.71
38	22.58	Benzene,1,1′-(1,2-dimethyl-1,2-ethenediyl)bis-, (z)-	C_16_H_16_	886	4.23
39	22.78	3,3,7,11-Tetramethyltricyclo[5.4.0.0(4,11)]undecan-1-ol	C_15_H_26_O	623	0.63
40	22.95	*(Z)*-Isomyristicin	C_11_H_12_O_3_	880	0.53
41	23.22	Spathulenol	C_15_H_24_O	783	0.41
42	23.36	Caryophyllene oxide	C_15_H_24_O	700	1.77
43	23.62	Carotol	C_15_H_26_O	830	0.66
44	24.01	*(E)*-Isomyristicin	C_11_H_12_O_3_	641	8.07
45	24.75	5,8-Methanonaphtho[2,3-b]furan-4,9-dione	C_12_H_6_O_3_	949	6.64
46	25.25	*α*-Eudesmol	C_15_H_26_O	762	0.52
47	25.57	*α*-Guaien	C_15_H_24_	867	0.72
48	25.79	*α*-Farnesene	C_15_H_24_	829	0.29
49	26.00	Spiro[4.5]decan-7-one	C_10_H_16_O	634	5.68
50	26.94	Tetradecanoic acid	C_14_H_28_O_2_	700	0.28
51	28.59	1,22-Docosanediol	C_22_H_46_O_2_	756	0.90
52	29.78	Methyl linolenate	C_19_H_32_O_2_	793	0.25
53	30.30	Methyl palmitate	C_17_H_34_O_2_	925	0.28
54	31.07	3,7-Dimethoxy-6h-dibenzo[b,d]pyran-6-one	C_15_H_12_O_4_	587	1.61
55	33.84	*(E)*-Phytol	C_20_H_40_O	870	1.12
56	34.36	Dehydroisophytol	C_20_H_38_O	681	0.39
57	36.45	Pentacosane	C_25_H_52_	352	1.50
58	36.68	Tetratetracontane	C_44_H_90_	785	2.18
59	40.23	Hexatriacontane	C_36_H_74_	506	0.54
60	43.28	Octacosane	C_28_H_58_	750	0.34
Total percentage of the relative area					99.99

**Table 2:** IC_50_ (µg/mL) of *A. orientale* EO using H_2_O_2_ and ABTS radical scavenging assays.

Sample	H_2_O_2_ Radical Scavenging IC_50_ (μg/mL)	ABTS Radical Scavenging IC_50_ (μg/mL)
*A. orientale* EO	10.44 ± 1.19 ^a^	8.51 ± 0.79 ^a^
Ascorbic acid	12.51 ± 0.56	13.65 ± 0.48

(^a^) *p* < 0.05, compared to ascorbic acid.

**Table 3:** IC_50_ (µg/mL) of *A. orientale* EO and doxorubicin against A549, HepG-2, HL-60, and MRC-5.

Sample	IC_50_ (μg/mL)			
	A549	HePG 2	HL-60	MRC-5
*A. orientale* EO	84.8	99.0	>100	9.31
Doxorubicin	28.3	21.6	45.6	26.1

**Table 4:** Apoptotic assay of *A. orientale* EO on lung cancer cell line (A549).

Sample	Tested Concentration (μg/mL)	Percent of Early Apoptosis	Percent of Late Apoptosis	Percent of Necrosis
*A. orientale* EO (Treated cells)	51.78	19.53	8.66	4.32
A549 cells (Control)	0	0.43	0.27	1.58

**Table 5:** Effect of *A. orientale* EO on A549 cell cycle.

Sample	Tested Conc. (μg/mL)	%G0-G1	%S	%G2-M
*A. orientale* EO (Treated cells)	84.8	46.53	34.85	18.62
A549 cells (Control)	0	51.04	36.15	12.81

**Table 6:** Effect of *A. orientale* EO on apoptotic protein levels (Bax, BCL2, caspase 3, and p53, as well as in Bax/Bcl2 ratios).

Samples		Protein Concentrations (μg/mL)			
	Bax	BCL2	Caspase-3	Bax/BCl2 (Ratio)	P-53
Control	4.19 ± 0.24	7.79 ± 0.32	59.41 ± 2.26	0.54 ± 0.03	3.91 ± 0.14
*A. orientale* EO	15.22 ± 0.24 ^a^	3.81 ± 0.24 ^a^	145.0 ± 3.58 ^a^	4.02 ± 0.38 ^a^	12.89 ± 0.84 ^a^

Data obtained from three replicates, expressed as the mean ± SD. The statistical significance of the results was indicated as (^a^) *p* < 0.05, related to the positive control.

**Table 7:** IC_50_ of *A. orientale* EO on the EGFR enzyme inhibition and *sorafenib*. The data, obtained from three replicates, are expressed as mean ± SD.

Sample	IC_50_ (μg/mL) A549
*A. orientale* EO	3.75 ± 0.63 ^a^
*Sorafenib*	0.77 ± 0.15

(^a^) *p* < 0.05, compared to the positive control.

**Table 8:** Docking scores of the binding interactions and the amino acids implicated in the binding of the identified compounds with the EGFR enzyme.

Compound Code	C-Docker Interaction Energy (Kcal mol^−1^)	Hydrogen Bond Interactions	Distance (Å)	Other Hydrophobic Interactions
W19 (Reference compound)	−58.14	MET793	3.78	CYS775, LEU777, ALA743 & LEU718, LEU858, LYS745 & VAL726
Myristicin	−33.36	ASP855	2.31	LEU792, LEU844& VAL726
		LYS745	2.76	
		THR854	2.28	
(Z)-Isomyristicin	−32.61	ASP855	2.25	LEU844, LYS745& VAL726
		THR854	2.30	
5,8-Methanonaphtho[2,3-b]furan-4,9-dione	−24.48	ASP855	2.40	MET766, LEU777, LEU788, LYS745, ALA743 & VAL726
		THR854	2.38	
*α*-Cedrene	−18.99	----	----	LEU844, VAL726, ALA743, LEU718, LEU1001 & PHE997
Spiro[4.5]decan-7-one	−28.70	ASP855	2.31	LEU788, VAL726, ALA743 & LEU858
7-Methylenebicyclo[4.2.0]oct2ene	−23.91	----	----	LEU788, LEU777 & LYS745
Ylangene	−22.85	----	----	LEU718, CYS797, LEU1001, VAL726 & PHE997
(*Z*)-but-2-ene-2,3-diyldi-benzene	−22.27	----	----	CYS797, LEU844, ALA743, LEU718 & VAL726
Alloocimene	−26.96	-----	-----	MET766, LEU788, LYS745, ALA743, PHE856, CYS775 & VAL726
5,7-Dimethylenebicyclo [2.2.2]oct-2-ene	−18.57	----	----	LEU777, LEU788, LYS745, PHE856 & CYS775
Camphene	−19.34	----	----	MET766, LEU777, LEU788, PHE856 & CYS775
Terpinolene	−29.68	-----	----	LEU777, LEU788, LYS745, PHE856, VAL726 & CYS775
Bicyclo[7.2.0]undec-3-ene,4,11,11-trimethyl-8 methylene-	−32.90	----	----	MET766, LEU777, LEU788, LEU858, LYS745, PHE856, LEU844 & VAL726
Caryophyllene oxide	−24.90	MET793	2.20	CYS797, LEU718, LEU844, PHE977, VAL726 & LEU1001

## Data Availability

The original contributions presented in this study are included in the article. Further inquiries can be directed to the corresponding author(s).
